# The effect of organic amendments on cannabidiol yield and topsoil health (0–10 cm) in floral hemp (*Cannabis sativa* L.) production system

**DOI:** 10.1186/s42238-026-00485-x

**Published:** 2026-08-14

**Authors:** Idowu Atoloye, Dipti Rai, Ashlee George, Pippin Massie, Arnab Bhowmik

**Affiliations:** 1 Department of Natural Resources and Environmental Design, North Carolina Agricultural and Technical State University, Greensboro, NC USA; 2 Department of Agriculture, Alcorn State University, Lorman, MS USA; 3 Department of Crop and Soil Sciences, North Carolina State University, Raleigh, NC USA

**Keywords:** Sustainable agriculture, Organic fertilization, Cover cropping, Soil biological activity

## Abstract

**Background:**

Compost and cover crops enhance soil health and crop productivity in agricultural systems, yet their integration as soil management strategies in floral hemp (*Cannabis sativa* L.) production remains underexplored. Understanding these effects is critical for optimizing cannabidiol yield while building soil health.

**Methods:**

In a two-year field study in north-central North Carolina, USA, we evaluated the effects of compost, cover crops, and their combination, and a non-fertilized control on soil health and cannabidiol yield in two floral hemp cultivars, Spectrum and Therapy. The experiment utilized a randomized complete block split-plot design with soil health indicators and agronomic parameters measured across multiple growth stages.

**Results:**

Treatment effects varied substantially by year and cultivar. In 2021, compost produced the highest cannabidiol yields for both cultivars–1.1 to 2.6 times greater than control and cover crop treatments. By 2022, compost-cover crop treatment had the highest yields for Spectrum, while Therapy responded minimally to amendments. Organic amendments increased soil organic carbon by 15%, total nitrogen by 20%, and microbial activity by 25%, particularly in year two. Mid-vegetative leaf nitrogen concentration emerged as a strong predictor of yield, explaining 52% of dry inflorescence and 40% of cannabidiol yield variation. Principal component analysis revealed that compost and compost-cover crop treatments clustered with enhanced soil biological indicators (soil organic carbon, total nitrogen, soil test biological activity, and permanganate oxidizable carbon) and higher yields, though year-specific environmental conditions resulted in distinct response patterns.

**Conclusions:**

Compost and combined compost-cover crop amendments improved soil health metrics, with benefits accumulating by year two. Cover crops alone provided minimal advantage. The effectiveness of organic amendments depends on cultivar selection and environmental conditions, necessitating multi-year evaluations to develop reliable management recommendations for CBD hemp production systems.

**Graphical Abstract:**

**Figure:**
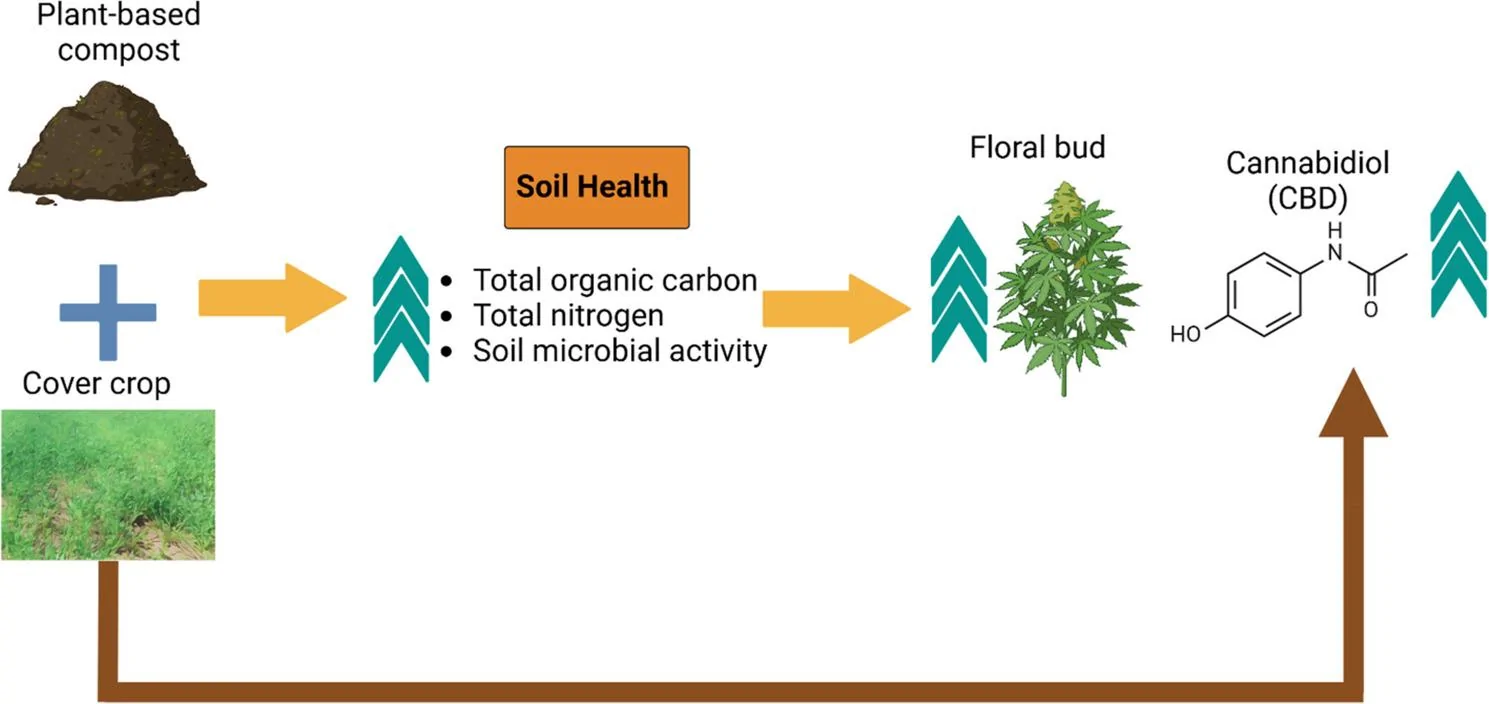

**Supplementary Information:**

The online version contains supplementary material available at https://doi.org/10.1186/s42238-026-00485-x.

## Introduction

Hemp (*Cannabis sativa* L.) has continued to gain significant attention in the United States due to the change in legislation surrounding its cultivation and production (Adesina et al. 2020; Ahmadi et al. 2024; Bruce et al. 2022). Hemp is known to serve many purposes and markets - from fiber, food, and oil production to medicinal uses (Adesina et al. 2020; Malone and Gomez 2019). Floral hemp refers to a specific subgroup of hemp varieties cultivated predominantly for their yield of floral biomass due to their high cannabinoid content (Linder et al. 2022). Since the lifting of the ban on hemp cultivation, published research has predominantly focused on inorganic fertilizers, greenhouse studies, and environmental stressors, with limited investigation into field practices such as green manures and organic amendments that could support long-term soil health enhancement in production systems (Farnisa et al. 2023; Linder et al. 2022; Tang et al. 2018; Westmoreland and Bugbee 2022).

Floral hemp is typically cultivated using plastic materials to control weeds, but this practice raises significant environmental concerns due to plastic waste accumulation and disposal challenges (Osorio et al. 2023). Traditional plastic mulch, while effective for weed suppression, contributes to agricultural pollution and requires costly removal and disposal after each growing season (Osorio et al. 2023). The reliance on plastic mulching systems also limits opportunities for building soil health through organic matter incorporation and beneficial microbial activity.

Despite the documented benefits of organic practices like cover crops and composting in agricultural systems (Chu et al. 2017; Thapa et al. 2018), it remains unclear whether their integration will affect hemp inflorescence and CBD yield. Even though a recent review showed that chemical fertilizers can increase hemp yields by up to 20% compared to organic alternatives (Ahmadi et al. 2024), emerging evidence suggests potential for organic approaches. A recent two-year field experiment in North Carolina, USA, on cannabinoid hemp production demonstrated that leguminous cover crops like hairy vetch can provide substantial nitrogen (N) contributions, with hairy vetch achieving 79% of the biomass yields compared to conventional nitrogen fertilization at 168 kg N ha^− 1^ (Moshay et al. 2024). Similarly, recent research on combined organic and inorganic fertilization strategies has shown promising results, where 50% replacement of mineral N with compost from solid digestate and cardoon-based spent mushroom substrate achieved comparable or even superior plant growth and biomass accumulation compared to conventional fertilization alone (Sicignano et al. 2025). Likewise, in a field experiment on a mesic Mollic Hapludalfs in Wisconsin, USA, compost (38 L per 0.93 m^2^ plot) application was shown to increase hemp biomass production, with greater flower, shoot, root, and total plant biomass compared to control treatments, while also improving soil health indicators such as pH, soil organic matter, and soil quality index (Desai et al. 2024). Thus, the combined use of both organic amendments can help reduce the reliance on mineral fertilizers (Chu et al. 2017), improving soil health conditions and often leading to enhanced yields over time, though the effects might not be immediately evident in the first year due to the gradual mineralization of certain biomass components (Geisseler et al. 2021; Martínez-Blanco et al. 2013). In addition, compost and cover crops differ in their N release patterns: leguminous cover crops typically provide faster nitrogen availability through rapid decomposition of their low C: N ratio biomass (C: N ratio of 10–25:1), while compost releases nutrients more gradually over time depending on its C: N ratio and degree of stabilization (Hunter et al. 2019). Composts with lower C: N ratios (15–25:1) release nitrogen more readily, whereas those with higher ratios (25–40:1) provide slower, sustained nutrient release as organic matter undergoes further decomposition and microbial processing (Marzi et al. 2020).

Furthermore, several studies have documented that the production of cannabinoids is strongly controlled by cultivar genetics, followed by harvest timing, and environmental conditions, with limited influence of fertilization practices (Bernstein et al. 2019; Crispim Massuela et al. 2022; Kalinowski et al. 2020; Linder et al. 2022). For example, Bernstein et al. (2019) observed that levels of tetrahydrocannabinol (THC) and CBD in the flowers remained unaffected by phosphorus (P) and NPK fertilizer treatments. Panday et al. (2025) demonstrated that varying rates of organic N fertilizer derived from bone meal affected CBD and THC concentrations. However, another study examining the impact of organic fertilization on cannabis suggested that excessive liquid organic fertilizer could potentially lead to reduced concentrations of THC in flowers as well as a decreased floral dry weight (Caplan et al. 2017). Similarly, in a pot experiment, Anderson et al. (2021) observed a decline in biomass production and a reduction in CBD concentration with fertigation rates greater than 300 ppm of N. These contrasting findings suggest that the relationship between nutrient management, biomass production, and cannabinoid concentration in hemp may be more complex than initially thought, potentially controlled by multiple factors like cultivar, growing conditions, and specific nutrient regimes.

Understanding the impact of compost and cover crops as a soil management strategy on floral hemp inflorescence yield is crucial for providing farmers with guidance to optimize CBD yield and improve soil health. In this study, we investigated the interactions of organic soil management practices on plant growth and cannabinoid production for two hemp varieties. We hypothesized that compost and cover crop treatments would improve soil health and hemp biomass, but acknowledged that environmental factors could mask treatment effects due to hemp’s photoperiodic sensitivity.

## Methods

### Experimental design

This research was conducted at the North Carolina Agricultural and Technical State University Farm (36°3′59′′N, 79°44′10′′W), Greensboro, North Carolina, USA. The soil type is Mecklenburg sandy clay loam (fine, mixed, active, thermic Ultic Hapludalfs) (USDA NRCS 2019). Baseline soil samples were collected and physically aggregated prior to the initiation of the experiment to characterize the initial soil physicochemical properties. Particle size was determined according to Gee and Bauder (1979) giving 58% sand, 13% silt, and 29% clay. Soil pH, 7.5, was measured in a 1:2 soil-to-water suspension, and cation exchange capacity (CEC), 11.2 cmol kg^− 1^, was determined by the ammonium acetate method (Chapman 1965). Total soil organic carbon (SOC) and total N were measured using a CHN analyzer (PerkinElmer, Akron, OH, USA; model 2400 Series II), with values of 12.3 g kg^− 1^ and 1.7 g kg^− 1^, respectively. Mehlich III P, 8.2 mg kg^− 1^, was determined colorimetrically (Sharpley et al. 1996), and nitrate-nitrogen, 1.2 mg kg^− 1^, was extracted with 2 M KCl and determined colorimetrically using a vanadium (III) assay (Doane and Horwáth 2003). The site is characterized by a humid subtropical climate. The total precipitation and mean air temperatures during the growing season (June through October) were 497 mm and 22.5 °C in 2021, and 485 mm and 21.7 °C in 2022 (Table S1) (State Climate Office of North Carolina, 2023).

The trial was arranged on a randomized complete block split-plot design with four replicates. The main plot effect was amendment: (1) compost (CM), (2) cover crop (CC), (3) compost and cover crop (CMCC), and (4) control (CL), and the subplot effect was variety. The amendments were applied to provide the recommended N rate of 100 kg N ha^− 1^ (Atoloye et al. 2022), with residual N of 12 kg N ha^− 1^ coming from terminated cover crops. The business-as-usual strategy for floral hemp N fertilization in North Carolina, United States, is 168 kg ha^− 1^ (Short et al. 2021). The CL treatment did not receive any N fertilizer, compost fertilization, or cover crops. The compost was a plant-based compost obtained from McGill AG (Cary, NC, USA), applied at rates of 100 kg N ha^− 1^ (72.2 Mt ha^− 1^ on a wet basis; moisture content of 46%) in the CM plot and 50 kg N ha^− 1^ in the CMCC plot. Compost was incorporated using a rototiller in the CM plots for both years, whereas in the CMCC plots, it was surface-applied in the first year and incorporated in the second. The compost contained (on a dry weight basis) total N of 25.3 g kg^− 1^, 19.2 g kg^− 1^ P, and 6.1 g kg^− 1^ K. Concentrations of other macronutrients included 6.1 g kg^− 1^ K, 71.6 g kg^− 1^ of Ca, and 2.8 g kg^− 1^ of Mg.

Cover crops, a mixture of crimson clover (*Trifolium incarnatum*) and cereal rye (*Secale cereale*), were planted in November at a seeding rate of 45 kg ha^− 1^ and 30 kg ha^− 1^, respectively, in the CC and CMCC plots (Table 1). The cover crops were terminated in April using a crimping roller and left as soil cover in 2021, but tilled into the soil’s top layer (0–10 cm) in 2022. This change was to promote better mineralization after the cover crop plots had low yields in 2021. Although the experiment was not conducted on a certified organic farm, hemp was managed in accordance with organic practices. No synthetic fertilizers or pesticides were applied during the crop cycle, and soil fertility inputs consisted solely of cover crops and compost incorporated prior to planting. Weed control was carried out either manually or mechanically throughout the growing season.

**Table 1:** Timing of field activities over a two-year period in the field study at the North Carolina Agricultural and Technical State University Farm near Greensboro, North Carolina, 2 USA

Field activities	2021	2022
*Cover crop*		
• Planting	Nov 16^*^	Nov 11^*^
• Termination via crimping	May 6	-
• Disking	May 27	May 15
• Roto tilling	May 27	May 15
• Re-crimping of cover crops	June 6	-
• Compost spreading and incorporation	May 27	May 28
*Hemp*		
• Delivery of Therapy clones	May 15	May 30
• Delivery of Spectrum Clones	May 15	May 30
• Beds and irrigation laid	June 7	June 6
• Planting of the Spectrum variety	June 8	June 15
• Planting of Therapy variety	June 8	June 24
• Re-plugging of dead plants	June 16, 24	-
*In-crop measurements*		
• Mid-vegetative stage data collection	July 7	July 12
• Late vegetative stage data collection	Aug 2	Aug 15
• Topping of plants	Sept 2	Sept 3
• First inflorescence sampling	-	Sept 8
• Pruning of lower branches	Sept 20	Sept 15
• Second inflorescence sampling	-	Sept 22
• Harvest – Therapy (except in cover crop plots)	-	Sept 24
• Third inflorescence sampling	-	Sept 29
• Harvest – Spectrum	-	Oct 6
• Harvest – Therapy (cover crop plots only)	-	Oct 12
• Harvest – Therapy	Oct 14	-
• Harvest – Spectrum	Oct 21	-

*The planting of cover crops occurred in the previous year

Two hemp varieties, Spectrum and Therapy, were acquired as six-week-old clones from certified seed stock, sourced from Carolina Canna, NC, and Old North State, NC, respectively. They were selected due to their low THC production and high inflorescence yield (Atoloye et al. 2022). The clones were transplanted to the field on June 8 in 2021, and on June 15 (Therapy) and June 24 (Spectrum) in 2022. The Clones were transplanted to a depth of 30 cm. The whole plot measured 6.86 m wide by 16.8 m long, while the sub-plots measured 3.43 m wide by 16.8 m long. Plants were spaced 1.5 m apart within rows and 1.8 m between rows, as there is no recommended spacing for CBD hemp. There were 10 plants in each sub-plot. Clones that died after transplanting were re-supplied within 2 weeks of initial planting. Planting dates of cover crops, hemp clones, and other field activities during the study are provided in Table 1. The plants were topped in the first week of September (approximately ten weeks post-transplant) and lower branches were pruned mid-September.

Irrigation lines were laid on the top of each bed, with two 3.8 L h^− 1^ emitters placed near the base of each plant on either side of the root system. Irrigation was applied three times per week, each morning for two hours, providing 15.2 L per plant per irrigation event, apart from when the soil was still moist from rain events. Over the 105-day growing season (June through mid-September), approximately 684 L of water from the experimental station dam was applied per plant. Any plants showing symptoms of southern stem blight (*Sclerotium rolfsii*) were carefully excavated from the field using disinfected tools to prevent disease spread.

### Plant measurements and analysis

Plant measurements were taken at three stages: mid-vegetative, late vegetative, and harvest on six out of the ten plants in each plot. The mid-vegetative stage was in mid-July, one month after transplanting, and characterized by vegetative growth with no indications of inflorescence. The late vegetative period, occurring in early August, was characterized by slowed vegetative growth, with inflorescences initiating on at least 50% of the field. Harvest was on October 14 and 21 in 2021 and October 6 and 12 in 2022, when the plants had been in floral bloom for 6–8 weeks and had more than 75% amber trichomes (Crispim Massuela et al. 2022). Plant height was measured to the nearest centimeter from the soil surface to the tallest point of the main shoot, with the meter stick kept perpendicular to the soil surface. The stem diameter was measured to the closest millimeter with calipers at the base of the stem, just above the soil surface. Canopy measurement was done by measuring across the plant’s widest point from an aerial view, then again perpendicular to the first measurement. Leaf length was measured from the leaf’s base (excluding the petiole) to the most extended tip, and width was measured from the two widest points of the leaf. Measurements from five leaves were taken from three randomly selected plants within each plot to ensure representative sampling. The third to fifth leaves from an axillary branch near the top of the plants were collected at mid- and late vegetative (McGinnis 2020). The leaves were rinsed with distilled deionized water and dried at 60 °C for 24 h. The dried leaf samples were ground using a Waring Commercial Blender (Conair LLC, Stanford, CT, USA) until homogenous and sent for nutrient analysis. Leaf nitrogen analysis was performed via dry combustion using a CHN analyzer 2400 series II (Perkin/2400 Elmer, Akron, OH, USA) by the Analytical Sciences Lab (North Carolina Agricultural and Technical State University, Greensboro, NC).

### Inflorescence harvest and cannabinoid analysis

In 2021, harvest occurred at 120 days after transplanting (DAT) for Therapy and 127 DAT for Spectrum when the plants had been in floral bloom for 6–8 weeks and had more than 75% amber trichomes (Crispim Massuela et al. 2022). However, in 2022, inflorescences were sampled at three stages (85, 99, and 105 DAT for Spectrum; 90, 99, and 118 DAT for Therapy) to determine the optimal harvest time by monitoring THC levels. After harvest, the inflorescences were manually trimmed, and fresh weights were recorded. The inflorescences were dried at 15–21 °C and 65% relative humidity in a drying chamber for 2 weeks until constant weight to obtain the dry inflorescence biomass. The average dry-weight biomass yield was measured per hectare using a plant density of 3,000 plants ha^− 1^. Composite fresh inflorescence samples from each field replicate were oven-dried at 60 °C for 48 h, then ground using a Waring Commercial Blender (Conair LLC, Stanford, CT, USA). Homogenized samples were then sent to Avazyme Inc (Durham, NC, USA) (2021) and Badger Lab (Stevens Point, WI, USA) (2022) for measuring CBD and THC via high-performance liquid chromatography (Agilent 1120 Infinity II, Santa Clara, CA, USA). Total CBD yield was calculated as dry inflorescence biomass × total CBD concentration, given the total weight of CBD produced per hectare.

### Soil health analysis

Soil samples were collected from the topsoil, 0–10 cm depth, using a soil core (i.d. 2.2 cm) at mid-vegetative (early-mid July) and late vegetative (early-mid August) stages. Three cores were taken near each plant base making a total of 18 cores per plot, composited, and transferred back to the lab. Soil gravimetric water content was measured by drying the field-moist soil at 105 °C until a constant weight was reached. Soil pH and electrical conductivity (EC) were determined using a soil-to-water ratio 1:2 (w/v). Total SOC and total N were analyzed using a CHN analyzer 2400 series II (Perkin/2400 Elmer, Akron, OH, USA). Nitrate nitrogen (NO_3_^−^-N) and ammonium nitrogen (NH_4_^+^-N) were determined from the filtered extract of sieved (≤ 2 mm) 5 g fresh soil (dry weight basis) that was shaken with 30 ml 2 M KCL for 30 min salicylate–nitroprusside and vanadium (III) using colorimetric assays according to Doane and Horwáth (2003) and Weatherburn (1967), respectively, using a Synergy HTX microplate reader (Biotek Instruments Inc, VT) at wavelengths of 540 and 650 nm, respectively. The potential soil dehydrogenase (DHA) enzyme activity was measured as described by Tabatabai (1994). Briefly, 2.5 g of fresh soil (dwb) was incubated with 0.5 mL of 3% triphenyl tetrazolium chloride (TTC), and 1.0 mL of 2% CaCO_3_ was added to each tube, vortexed, and incubated for 24 h at 37˚C. Thereafter, triphenyl formazan (product of TTC reduction) was extracted using 10 mL of methanol and measured colorimetrically with a microplate reader at a wavelength of 490 nm.

A combined assay for the simultaneous determination of four soil enzymes, β-glucosidase, β-glucosaminidase, acid phosphatase, and arylsulfatase (CNPS), was performed as described by Acosta-Martínez et al. (2019). Briefly, 0.5 g of air-dried soil was incubated with 0.5 mL of buffer (acetate buffer) and 2 mL of the solution with the four substrates (0.5 mL of each substrate prepared in the same buffer used in the assay) for 1 h at 37 °C. Following incubation, 0.5 mL of 1.0 M CaCl_2_ was added, followed by 2 mL of 0.1 M Tris(hydroxymethyl) aminomethane, pH 12.0, and the color was determined colorimetrically at 400 nm.

Soil permanganate oxidizable carbon (POXC) was measured using colorimetric analysis at 550 nm with a microplate reader as described by Weil et al. (2003). 2.5 g of air-dry soil was shaken with 18 mL of deionized water and 2 mL of 0.2 M potassium permanganate (KMnO_4_) solution. The samples were placed on an end-to-end shaker for 2 min at 240 oscillations per minute, followed by a 10-minute settling period. Subsequently, 0.5 mL of the clear supernatant was diluted with 49.5 mL of deionized water. Aliquots of 200 µL from each diluted sample were then transferred to a 96-well microplate alongside KMnO_4_ standards for absorbance measurement at 550 nm. Soil test biological activity (STBA) (Franzluebbers and Assmann 2020) was assessed using a LI-830 CO2 gas analyzer (LICOR Biosciences, NE). STBA was determined from the flush of CO_2_ following rewetting of dried soil with aerobic incubation of soil at 50% water-filled pore space and 25 °C. Exactly 50 g soil samples were placed in 60-ml glass jars, wetted, and placed in a 1-L canning jar and placed in a temperature-controlled humidity chamber. At the end of day three of the incubation, 0.5 mL of CO_2_ gas was collected and analyzed.

### Statistics

Statistical analysis was performed using generalized linear mixed-effects models with amendment and variety as fixed effect factors, block as a random effect, and year as a repeated measure (SAS v9.4 University Edition, Cary, NC). The mean comparison was carried out using the adjusted Tukey’s method at *p* = 0.05. Where the *F*-statistic was not significant, a simple effect comparison of the interaction (LSMESTIMATE) was done. Data were analyzed for each stage separately. The data were assessed to ensure they met the normality and constant variance assumptions and log-transformed if needed. Principal Component Analysis (PCA) was performed to explore the relationships between soil variables and plant variables using “ggplot2” package (Wickham 2011). Subsequently, Pearson’s product-moment correlation coefficients between soil properties and plant characteristics were calculated using the “ggcorrplot” package. Graphs were made using R version 4.0.5 (R Core Team 2025).

## Results

### Environmental conditions

Weather conditions varied considerably across the two growing seasons (Table S1) (State Climate Office of North Carolina, 2023). During 2021, precipitation from June through October totaled 497 mm, while 2022 received slightly less at 485 mm over the same period - but both were close to the 30-year normal of approximately 486 mm (National Weather Service 2025). However, June 2021 was notably wetter (97 mm) compared to June 2022 (48 mm). Precipitation in June 2021 was closer to the 30-year average (93 mm), while 2022 was notably drier. Conversely, in 2022, precipitation was substantially higher in July (177 mm) than in 2021 (133 mm). Late-season precipitation patterns were relatively similar between years, with September and October receiving between 71 and 93 mm monthly. Average temperatures during the hemp growing season showed consistent patterns across both years and is close to the 30-year normals (approximately 24–26 °C) (National Weather Service 2025). Peak summer temperatures (June-August) averaged 23–25 °C. Temperatures declined toward harvest, reaching approximately 17 °C in October 2021 and 14 °C in October 2022.

### Inflorescence and CBD yield data

**Fig. 1:**
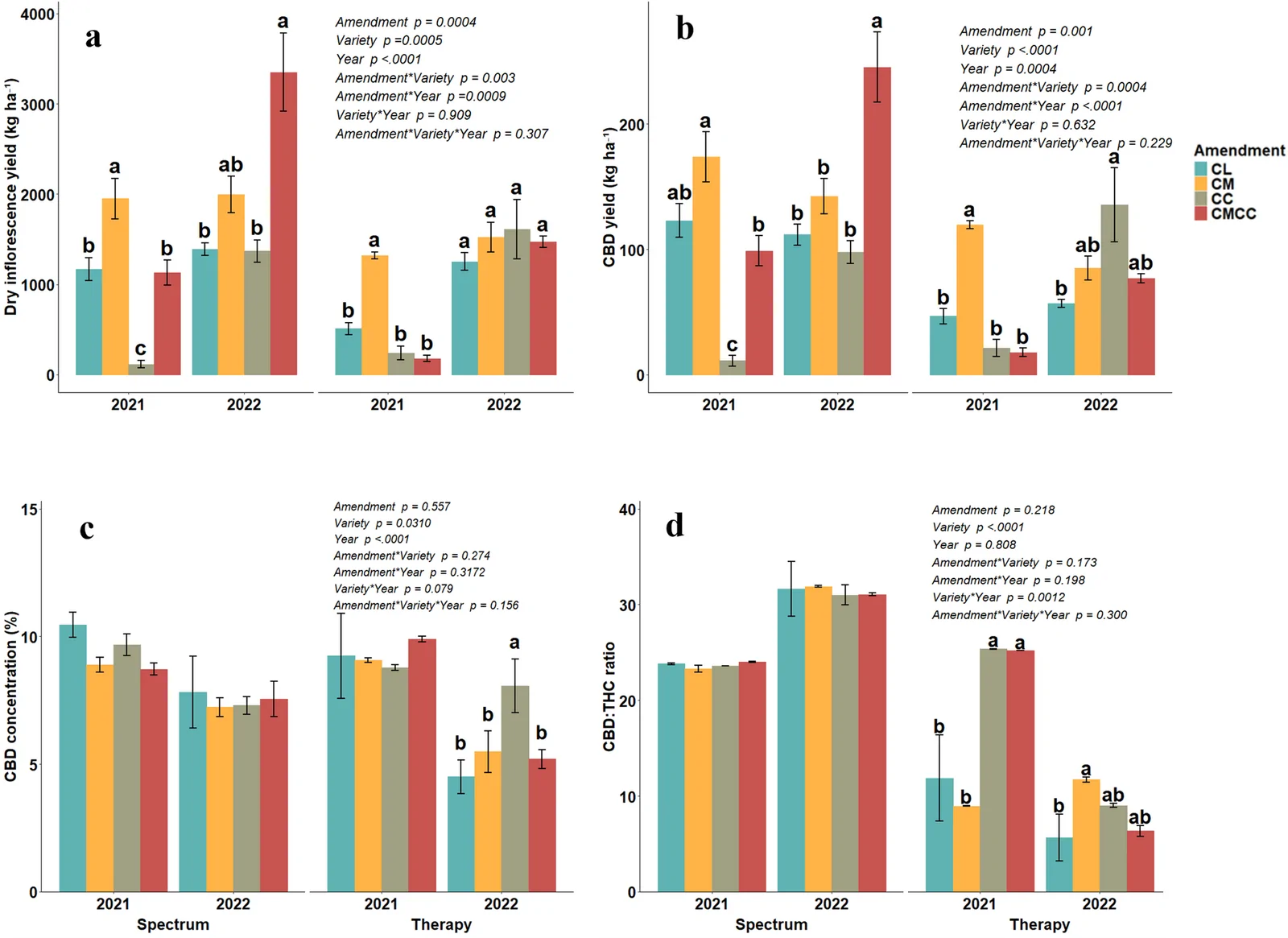
Effect of amendments on (**a**) dry inflorescence yield, **b** cannabidiol (CBD) yield, **c** CBD concentration, and (**d**) tetrahydrocannabinol (THC) concentration in hemp during 2021 and 2022. Error bars represent standard errors (n = 3). CL = control; CM = compost; CC = cover crop only; CMCC = compost and cover crop. Mean separation was based on simple-effect comparisons of the interaction (LSMEANS estimate). Different letters indicate significant differences among amendments within the same year at *p* < 0.05

Dry inflorescence yield varied across amendments, variety, and year (Figs. 1a). In 2021, dry inflorescence yield for both varieties was highest under CM (1972 kg ha^− 1^ for Spectrum and 1421 kg ha^− 1^ for Therapy). However, in 2022, CMCC treatment produced the highest dry inflorescence yield for Spectrum (3500 kg ha^− 1^), while Therapy showed no significant differences among amendments, with yields ranging from 1200 to 1600 kg ha^− 1^ across all treatments. Similarly, differences in CBD yield mirrored the effects of amendments, variety, and year on dry inflorescence yield, except for the CBD yield of Therapy in 2022 (Fig. 1b). While the dry inflorescence yield of Therapy was not significantly different, the CBD yield under CC was significantly higher than CL. Overall, dry inflorescence and CBD yield were consistently higher for Spectrum than the Therapy variety. CBD concentration among the treatments was only significantly different for Therapy variety in 2022 (Fig. 1c). The CBD percent concentration for Therapy variety in 2022 followed the trend CC > CM = CMCC = CL. The CBD: THC ratio showed significant effects of variety (*p* = 0.031) and year (*p* < 0.0001), but no significant effect of amendment (*p* = 0.557) (Fig. 1d). It is important to know that the THC values were generally above the legal threshold of 0.3% (Table S2). For Spectrum variety, the CBD: THC ratio remained relatively stable across both years and all amendments, with no significant differences among treatments in either year. In contrast, Therapy variety exhibited more variable responses. In 2021, CC and CMCC treatments showed significantly higher ratios compared to CL and CM. In 2022, the CBD: THC ratio of CM was significantly higher than CL, but not CC and CMCC.

### Vegetative growth

The three-way interaction (amendment × year × variety) was not significant at the mid-vegetative stage (*p* = 0.156) (Table 2). This interaction was significant at late vegetative stage (F = 7.67, *p* < 0.0001), indicating that cultivar-specific responses to organic amendments were environmentally dependent and that variety-specific treatment effects became more pronounced as plants matured (Table 2). In 2021, Therapy height varied significantly among the amendments, ranging from 33 cm under CC to 78 cm under CL. Spectrum maintained more consistent height across treatments in 2021 (69–121 cm). Variety remained the dominant factor controlling plant height at both stages (*p* < 0.0001), with Spectrum consistently taller than Therapy. Also, in 2022, following cover crop incorporation in the CC and CMCC plots, both cultivars performed well.

**Table 2:** Effect of amendments on plant height and base stem diameter

		Plant height (cm)				Base stem diameter (mm)			
		MV		LV		MV		LV	
		2021	2022	2021	2022	2021	2022	2021	2022
Spectrum	CL	51(4)a	46(4)ab	82(6)bc	108(8)	17(1)b	7(0)b	20(1)b	21(1)c
	CM	58(4)a	39(3)b	121(8)a	114(9)	19(1)a	7(0)b	27(1)a	25(1)b
	CC	38(3)b	49(4)ab	68(5)c	122(9)	14(1)c	7(0)b	16(1)c	25(1)b
	CMCC	53(4)a	57(5)a	89(6)b	125(10)	17(1)b	10(1)a	20(1)b	28(1)a
Therapy	CL	28(2)ab	22(2)b	75(5)a	82(6)b	15(1)b	3(0)	22(1)a	14(1)c
	CM	31(2)a	28(2)a	73(5)a	103(8)a	17(1)a	4(0)	23(1)a	20(1)b
	CC	18(1)c	29(2)a	32(2)c	101(8)ab	13(1)c	3(0)	14(0)b	18(1)bc
	CMCC	22(1)bc	28(2)a	40(2)b	111(8)a	14(1)bc	4(0)	16(1)b	23(1)a

The data represent the mean (standard error); Different lowercase letters indicate significant differences among amendments within the same stage (linear mixed-effect model and Tukey’s HSD; *n* = 3). The mean separation was performed separately for each species and using a simple effect comparison of the interaction (LSMESTIMATE)

*MV* mid-vegetative, *LV* late-vegetative, *CL* control, *CM* compost, *CC* cover crop only, *CMCC* compost and cover crop

Similarly, base stem diameter responses varied by variety, year, and growth stage (Table 2). In 2021, or Spectrum in 2021, CM treatment consistently produced the largest stem diameter at both mid-vegetative (19 mm) and late vegetative (27 mm) stages, while CC had the smallest diameter. In 2022, CMCC produced the largest stem diameter for Spectrum at both stages (10 mm at mid-vegetative and 28 mm at late vegetative). For Therapy, CM produced the largest stem diameter at the mid-vegetative stage in 2021 (17 mm), though at the late vegetative stage, CM and CL were not significantly different. In 2022, no significant differences were observed among treatments at the mid-vegetative stage for Therapy, but at the late vegetative stage, CMCC produced the largest stem diameter (23 mm), while CL had the smallest (14 mm).

Leaf N response to amendments were limited across the varieties and stages (Table 3). In 2021, CM consistently produced the highest leaf N concentration for both varieties at both growth stages (4.87–5.25% for Spectrum and 4.51–4.78% for Therapy). While CC produced the lowest leaf N concentration at the mid-vegetative stage, there was no significant difference between leaf N in plants under CC versus CL and CMCC by the late vegetative stage. However, in 2022, no significant differences in leaf N concentration were observed among treatments for either variety at both growth stages, with values ranging from 4.40 to 5.53% for Spectrum and 4.25–5.26% for Therapy.

**Table 3:** Effect of amendments on plant characteristics in 2021 and 2022

	Leaf *N* (%)							
	MV				LV			
	SP		TH		SP		TH	
Amendment	2021	2022	2021	2022	2021	2022	2021	2022
CL	4.0 (0.2)b	5.2 (0.2)	3.4 (0.2)b	5.0 (0.2)	4.1 (0.2)b	4.5 (0.3)	3.7 (0.2)b	4.3 (0.2)
CM	5.3 (0.2)a	5.3 (0.2)	4.9 (0.2)a	5.3 (0.2)	4.9 (0.3)a	5.1 (0.3)	4.5 (0.2)a	4.5 (0.3)
CC	3.2 (0.1)c	5.4 (0.2)	2.7 (0.1)c	5.3 (0.2)	3.7 (0.2)b	4.7 (0.3)	3.3 (0.2)b	4.4 (0.2)
CMCC	3.8 (0.2)b	5.5 (0.3)	3.3 (0.2)bc	5.2 (0.2)	4.1 (0.2)b	4.4 (0.2)	3.2 (0.2)b	4.8 (0.3)
	Leaf area (cm^2^)							
CL	-	79(10)ab	-	41(5)	45(4)	59(5)	47(6)a	42(3)
CM	-	72(9)b	-	38(6)	50(3)	61(6)	41(3)ab	45(4)
CC	-	93(10)a	-	41(4)	52(4)	74(5)	29(3)b	38(3)
CMCC	-	89(10)a	-	40(4)	47(4)	65(5)	36(5)ab	53(5)
	Canopy area (m^2^)							
CL	-	0.15(0.02)bc	-	0.03(0.00)b	-	0.82(0.05)	-	0.45(0.05)b
CM	-	0.12(0.03)c	-	0.05(0.01)a	-	0.89(0.12)	-	0.71(0.03)a
CC	-	0.17(0.02)b	-	0.05(0.01)a	-	0.97(0.09)	-	0.62(0.04)ab
CMCC	-	0.29(0.03)a	-	0.05(0.00)a	-	1.16(0.07)	-	0.94(0.08)a

The data represent the mean (standard error); Different lowercase letters indicate significant differences among amendments within the same stage (linear mixed-effect model and Tukey’s HSD; *n* = 3). The mean separation was performed separately for each species and using a simple effect comparison of the interaction (LSMESTIMATE)

*MV* mid-vegetative, *LV* late-vegetative, *CL* control, *CM* compost, *CC* cover crop only, *CMCC* compost and cover crop

Leaf area showed limited treatment effects (Table 3). There was no statistical difference between the control and other treatments, although CM differed significantly from CC and CMCC for Spectrum in 2021 only. Plant canopy area was measured in 2022 only (Table 3). Significant main effects were observed for the main effects, i.e., amendment (*p* = 0.002), variety (*p* < 0.001), and stage (*p* < 0.001), and their interaction was also significant (*p* = 0.007). At the mid-vegetative stage, Spectrum plants under CMCC had the highest canopy area (0.29 m^2^), which was significantly greater than all other treatments, while CM produced the smallest canopy (0.12 m^2^) but this was not different from CL. For Therapy, there was no significant difference among CM, CC, and CMCC treatments (all 0.05 m^2^), with the lowest canopy measured under CL (0.03 m^2^). However, at the late vegetative stage in 2022, no significant differences in canopy area were observed among treatments for Spectrum, with values ranging from 0.82 to 1.16 m^2^. For Therapy, CM and CMCC produced the largest canopy areas (0.71 and 0.94 m^2^, respectively), which were significantly greater than CL (0.45 m^2^), but not CC.

### Soil health and biochemical properties

The main effect of amendments was significant (*p* < 0.05) for SOC and TN across both years (Table 4). SOC was highest in the CM treatment in 2021 and 2022. Furthermore, SOC in the CMCC treatment was significantly higher than in CC and CL in 2022 but not in 2021. There was no significant difference in SOC between CL and CC treatments. Soil TN was consistently highest in the CM treatment, followed by CMCC, which had higher TN values than CC in 2022 (*p* < 0.037) but not in 2021 (*p* < 0.089). No differences in soil TN were observed between the CL and CC treatments in either year. Relative to the baseline of 18.1 Mg ha⁻¹, estimated SOC stocks increased under all treatments by 2022, but the magnitude depended on amendment (Table 5). CM plots nearly doubled SOC stocks over the two years, while CC and the control CL plots increased only marginally (~ 15%). The overall SOC accrual rate under CM (7.38 Mg ha^− 1^ yr^− 1^) was roughly five times that of CC and CL, with CMCC intermediate (*p* = 0.001). The two compost treatments differed in timing: CM accrued most rapidly in the first year and then slowed, whereas CMCC accelerated, reaching the highest second-year rate of any treatment, though the 2021–2022 rates were not statistically significant (*p* = 0.142). Total N followed the same ranking, with CM showing the largest overall gain (~ 60% above baseline) and the highest accrual rate (*p* = 0.094).

**Table 4:** Effect of amendments on soil organic carbon (SOC), total nitrogen (TN), ammonium (NH_4_-N), and nitrate nitrogen (NO_3_-N) and the levels of significance of F tests from the ANOVA

	MV				LV			
	SOC	TN	NH_4_-*N*	NO_3_-*N*	SOC	TN	NH_4_-*N*	NO_3_-*N*
Amendment	(g kg^− 1^)		(mg kg^− 1^)		(g kg^− 1^)		(mg kg^− 1^)	
CL	14.5 (1.3)ab	1.87 (0.2)b	17.1 (3.6)	11.6 (1.3)b	14.3 (0.6)c	1.95 (0.1)b	18.7 (2.3)b	13.7 (1.9)b
CM	19.4 (1.8)a	2.47 (0.2)a	17.7 (3.7)	46.1 (5.1)a	21.5 (0.9)a	2.53 (0.1)a	24.6 (3.1)ab	24.4 (3.4)a
CC	14.5 (1.3)b	1.82 (0.2)b	23.9 (5.0)	7.8 (0.9)c	13.2 (0.5)c	1.92 (0.1)b	28.7 (3.6)a	16.2 (2.3)ab
CMCC	16.8 (1.5)ab	2.06 (0.2)ab	20.3 (4.3)	10.3 (1.2)bc	17.0 (0.7)b	2.37 (0.1)b	31.3 (3.9)a	24.3 (3.4)a
Year								
2021	15.0 (0.8)b	1.87 (0.1)b	13.2 (2.1)b	10.2 (0.8)b	15.2 (0.4)b	2.00 (0.1)b	47.3 (4.9)a	22.1 (2.2)a
2022	17.5 (1.0)a	2.23 (0.2)a	29.0 (4.5)a	20.3 (1.6)a	17.3 (0.5)a	2.36 (0.1)a	13.6 (1.4)b	16.4 (1.6)b
Effect	F Tests from the ANOVA							
Amendment (A)	0.129	0.093	0.550	0.000	0.001	0.015	0.040	0.059
Variety (V)	0.612	0.732	0.872	0.627	0.797	0.708	0.166	0.564
Year (Y)	0.022	0.003	< 0.0001	< 0.0001	0.004	0.003	< 0.0001	0.050
A*V	0.766	0.751	0.997	0.564	0.387	0.508	0.976	0.492
A*Y	0.568	0.693	0.099	< 0.0001	0.423	0.951	0.011	0.003
V*Y	0.445	0.676	0.647	0.314	0.277	0.121	0.791	0.190
A*V*Y	0.899	0.528	0.857	0.530	0.137	0.504	0.560	0.447

The data represent the mean (standard error); Different lowercase letters indicate significant differences among amendments within the same stage (linear mixed-effect model and Tukey’s HSD; *n* = 3). The mean separation was performed separately for each species and using a simple effect comparison of the interaction (LSMESTIMATE)

*MV* mid-vegetative and *LV* late-vegetative, *CL* control, *CM* compost, *CC* cover crop only, *CMCC* compost and cover crop

**Table 5:** Estimated soil organic carbon (SOC) and total nitrogen (TN) stocks and accrual rates under different organic soil amendments at 0–10 cm depth

	SOC			Accrual rate			TN			Accrual rate		
	Mg ha^− 1^			Mg ha^− 1^ yr^− 1^			Mg ha^− 1^			Mg ha^− 1^ yr^− 1^		
	Baseline^*^	2021	2022	Baseline − 2021	2021–2022	Overall baseline − 2022	Baseline	2021	2022	Baseline − 2021	2021–2022	Overall baseline − 2022
CL	18.1 ± 0.2^†^	20.1 ± 1.1b^‡^	20.9 ± 0.9c	2.08 ± 0.6b	0.86 ± 1.3b	1.57 ± 0.5c	2.50 ± 0.1	2.51 ± 0.1c	3.16 ± 0.2b	0.07 ± 0.1c	0.65 ± 0.2	0.34 ± 0.1b
CM	18.1 ± 0.2	27.7 ± 1.6a	32.6 ± 1.4a	10.8 ± 2.4a	4.92 ± 2.6ab	7.38 ± 0.6a	2.50 ± 0.1	3.36 ± 0.2a	4.09 ± 0.2a	0.89 ± 0.2a	0.74 ± 0.3	0.81 ± 0.1a
CC	18.1 ± 0.2	19.7 ± 1.1b	21.0 ± 0.9c	1.8 ± 0.9b	1.37 ± 1.5b	1.59 ± 0.5c	2.50 ± 0.1	2.62 ± 0.1bc	2.87 ± 0.2b	0.13 ± 0.1bc	0.25 ± 0.2	0.24 ± 0.1b
CMCC	18.1 ± 0.2	22.1 ± 1.2b	28.2 ± 1.2b	4.01 ± 0.6b	6.09 ± 1.2a	5.05 ± 0.5b	2.50 ± 0.1	2.71 ± 0.1b	3.48 ± 0.2ab	0.48 ± 0.1ab	0.77 ± 0.2	0.56 ± 0.1ab
*p* -value		0.003	0.001	0.001	0.142	0.001		0.002	0.001	0.007	0.713	0.094

*CL* control, *CM* compost, *CC* cover crop only, *CMCC* compost and cover crop

* Value excluded from statistical analysis. Samples for baseline were physically aggregated prior to analysis

†Values were estimated using an approximate bulk density of 1.47 g cm^− 3^ obtained from Web Soil Survey for the Mecklenburg sandy clay loam series

^‡^ Means in the same column with similar lower-case letters are not significantly different using LSMEANS separation for multiple comparison

Soil NH_4_^+^-N and NO_3_^−^-N exhibited significant changes with amendment (*p* < 0.05) (Table 4). In 2021, CMCC plots had significantly higher NH_4_^+^-N levels for both varieties at the late vegetative stage. In 2022, there were no statistical differences in NH_4_^+^-N levels under the Therapy variety, but Spectrum CM treatment had the highest NH_4_^+^-N levels. Soil NO_3_^−^-N levels were highest under CM (46 mg kg^− 1^) at the mid-vegetative stage, while at the late vegetative stage, it was not significantly different between CM and CMCC.

**Fig. 2:**
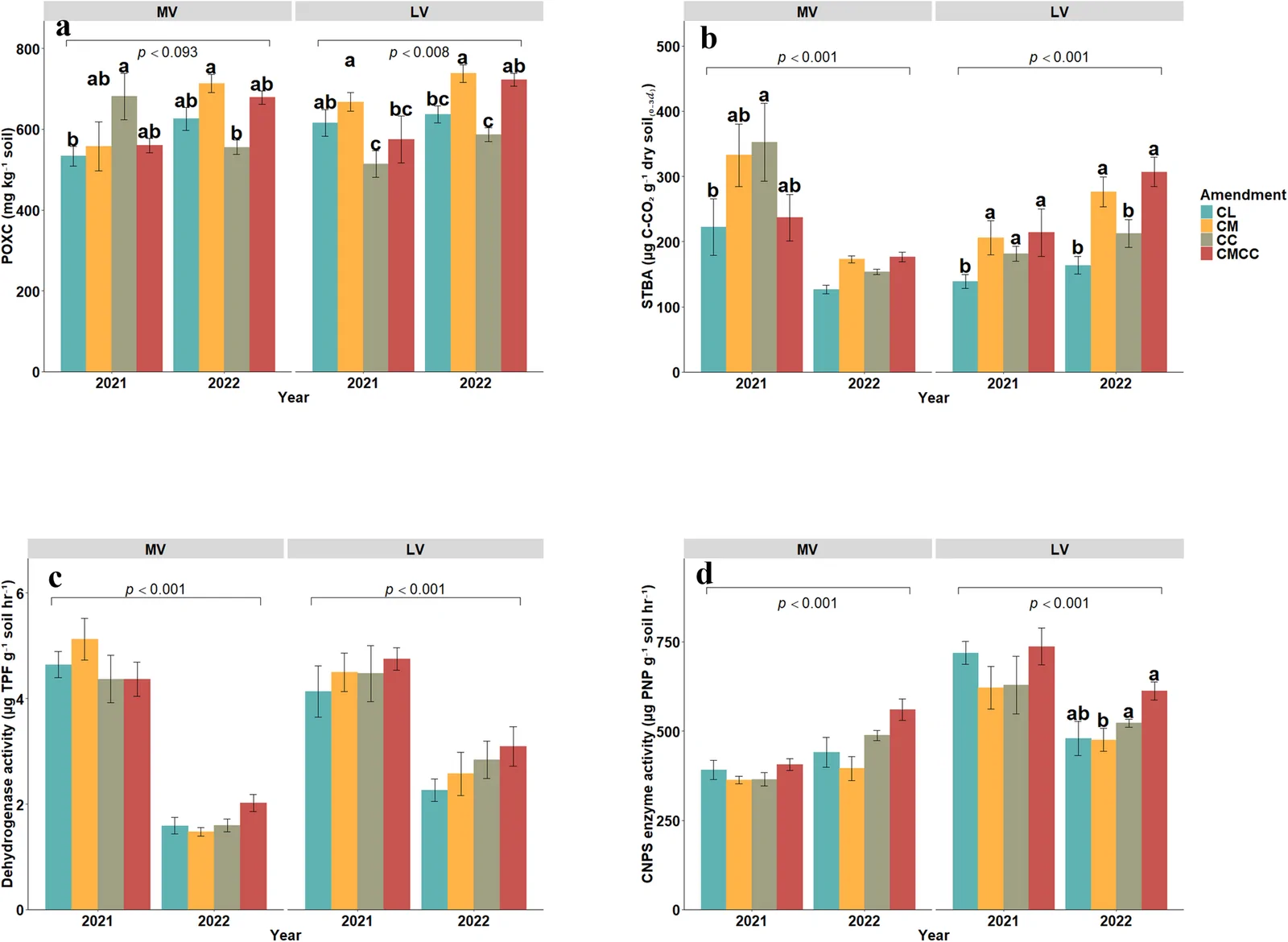
Effect of amendments on (**a**) permanganate oxidizable carbon (POXC), **b** soil-test biological activity (STBA), **c** dehydrogenase enzyme activity, and (d) combined carbon, nitrogen, phosphorus, and sulfur enzymes activities (CNPS) averaged across variety. Error bars represent the standard errors (n = 3). CL, control; CM, compost; CC, cover crop only; CMCC, compost and cover crop. Mean separation was based on a simple-effect comparison of the interaction (LSMESTIMATE). Different letters indicate significant differences among amendments within the same year at *p* < 0.05

The main effect of amendments was significant (*p* < 0.05) for measured POXC, STBA, and CNPS in 2022 but not in 2021 (Fig. 2a, b, and d). The LSMEANS analysis further elucidated the differences among treatments, revealing significant pairwise differences. In 2021, POXC was higher in CM in Spectrum plots at the late vegetative stage, whose biomass is higher than that of Therapy, and in 2022, it was greater in CM and CMCC plots after the incorporation of cover crop into the soil. The STBA was only significant in 2021 with Therapy variety, while CM and CMCC plots showed greater STBA in 2022 for both varieties. In 2021, CNPS was greater in CMCC and CL plots. Meanwhile, in 2022, CMCC showed higher CNPS activity. DHA was not significantly different among the treatment or growth stage except for between years (Fig. 2c).

Soil pH and EC were significantly affected by soil amendments (*p* < 0.001) across both growing seasons (Table S3). The CM treatment consistently reduced soil pH (6.9–7.2) compared to the CL (7.5–7.6) for both varieties at most growth stages, likely due to organic acid production during compost decomposition. Correspondingly, compost treatments exhibited substantially higher EC values (154–531 µS cm^− 1^) compared to other treatments, with the most pronounced differences observed at mid-vegetative stages.

### Correlation and principal component analysis

**Fig. 3:**
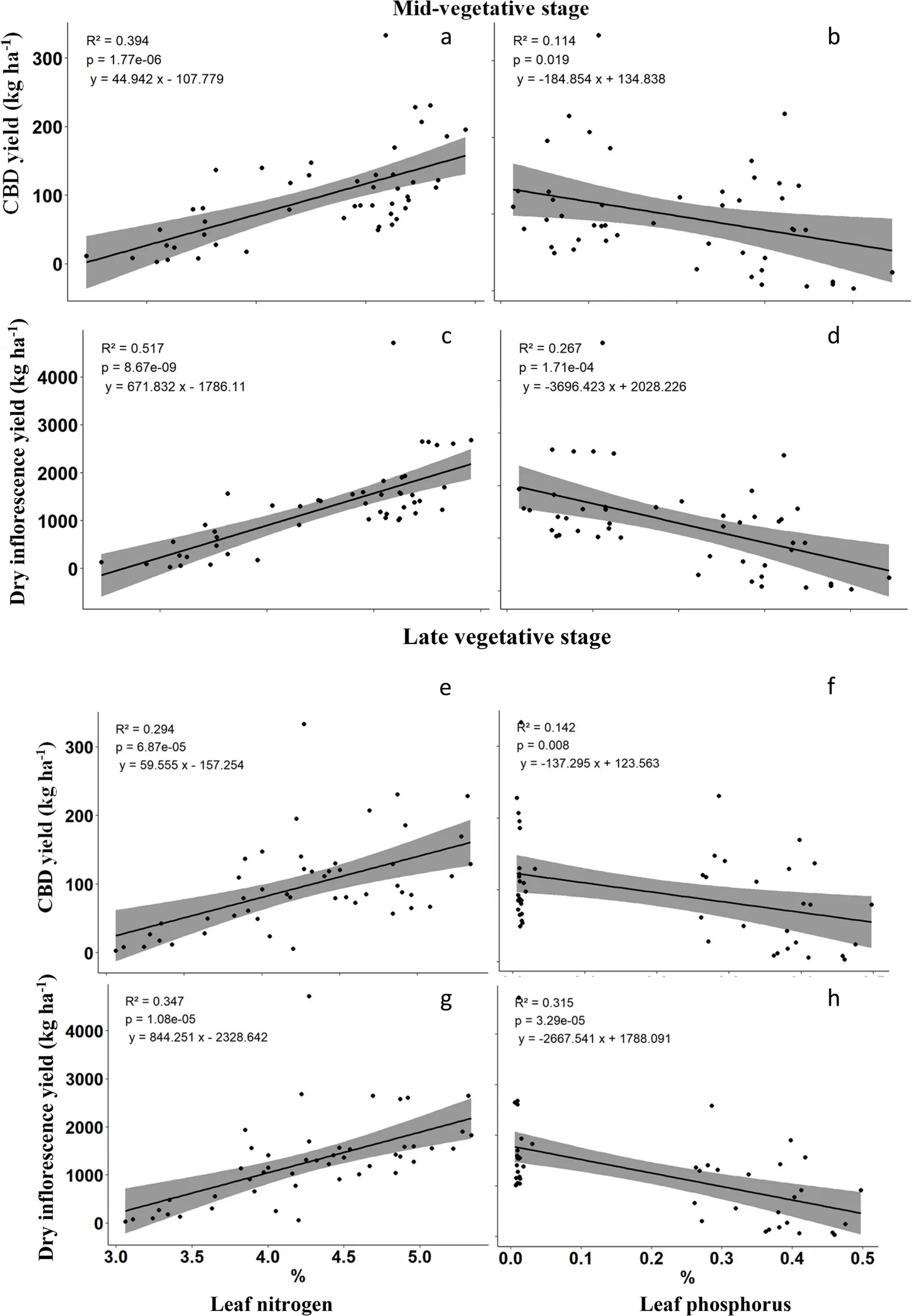
Pearson correlation between leaf nutrient concentrations and yield parameters across years and varieties. Panels (**a**) and (**e**) show the relationship between leaf nitrogen concentration and cannabidiol (CBD) yield at the mid-vegetative and late-vegetative stages, respectively; panels (**b**) and (**f**) show the relationship between leaf phosphorus concentration and CBD yield at the mid-vegetative and late-vegetative stages, respectively; panels (**c**) and (**g**) show the relationship between leaf nitrogen concentration and dry inflorescence yield at the mid-vegetative and late-vegetative stages, respectively; and panels (**d**) and (**h**) show the relationship between leaf phosphorus concentration and dry inflorescence yield at the mid-vegetative and late-vegetative stages, respectively. Stronger associations were observed at the mid-vegetative stage than at the late-vegetative stage. Solid lines represent linear regression fits, and shaded areas indicate 95% confidence intervals

Regression analysis revealed that leaf N concentration was positively correlated with both dry inflorescence yield and CBD yield at both vegetative stages, with stronger predictive power at the mid-vegetative stage where it explained 52% of dry inflorescence yield variation and 40% of CBD yield variation (*p* < 0.001) compared to 34.7% and 29.4% respectively at the late vegetative stage (*p* < 0.001). Conversely, leaf P concentration showed negative correlations with both yield parameters, explaining 23.5% of dry inflorescence yield variation (*p* < 0.001) and 8.7% of CBD yield variation (*p* = 0.047) at mid-vegetative stage, with explanatory power increasing to 31.5% (*p* < 0.001) and 14.2% (*p* = 0.008) respectively at the late vegetative stage (Fig. 3a-h).

The correlation analysis among soil health indicators and agronomic parameters showed a significantly stronger relationship between soil and plant parameters in 2021 than in 2022 (Figure S1a-d). At the mid-vegetative and late vegetative stages in 2021, several indicators strongly correlated with both CBD and dry inflorescence yields. However, in 2022, DHA and STBA at the mid-vegetative stage and STBA, TOC, and TN at the late vegetative stage were significantly correlated with the CBD yield, dry inflorescence yield, and other phenotypical parameters. This shift in indicator relationships suggests improvement in soil health in the second year.

**Fig. 4:**
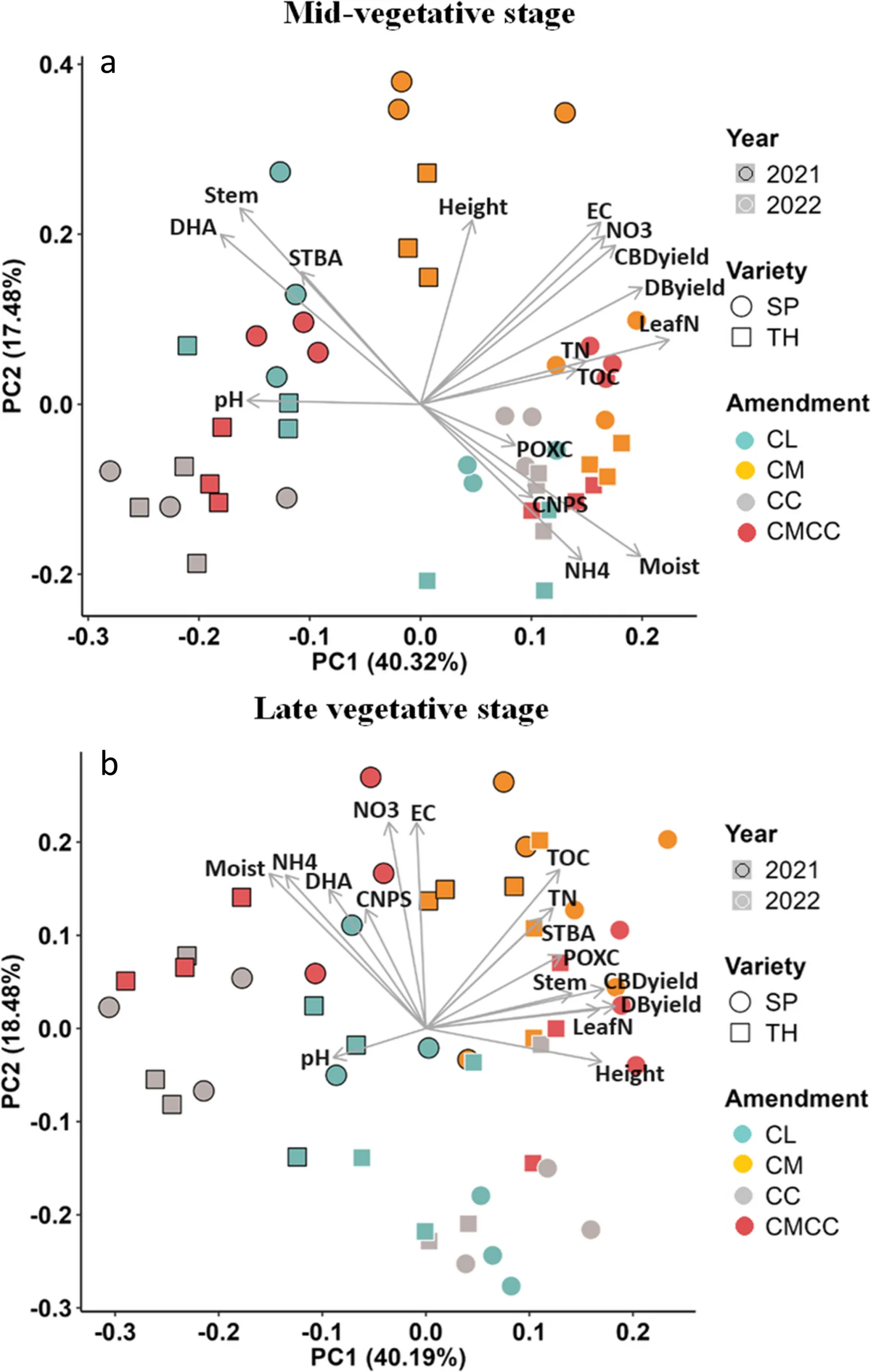
Principal component analysis (PCA) showing plant and soil properties at the (**a**) mid-vegetative stage and (**b**) late-vegetative stage. Each point represents an individual sample collected from the experimental site. CL, control; CM, compost; CC, cover crop only; CMCC, compost and cover crop; SP, Spectrum; TH, Therapy; CBD, cannabidiol; DB, dry bud (dry inflorescence) yield; N, nitrogen; Moist, gravimetric moisture content; EC, electrical conductivity; TOC, total organic carbon; TN, total nitrogen; NO₃, nitrate; NH₄, ammonium; POXC, permanganate oxidizable carbon; STBA, soil-test biological activity; DHA, dehydrogenase enzyme activity; CNPS, combined carbon, nitrogen, phosphorus, and sulfur enzymes activities

Principal component analysis showed relationships between soil amendments, soil health, and hemp productivity across growth stages (Fig. 4a-b). At the mid-vegetative stage, the first two components explained 57.72% of variance, with soil chemical indicators (NO₃⁻-N and electrical conductivity) correlating with yield parameters. At the late vegetative stage, components explained 59% of variance, with CM and CMCC treatments grouping with biological soil health indicators (TOC, TN, STBA, POXC) and higher hemp performance. Leaf N consistently predicted both dry inflorescence and CBD yields across growth stages, while organic amendments (CM and CMCC) associated with improved soil biological activity rather than chemical fertility alone. Data points were separated by year, indicating that environmental factors affected treatment responses (Khatri et al. 2025).

## Discussion

### Amendment effect on dry inflorescence and CBD yield

While a number of studies have examined the effect of mineral fertilizers on hemp inflorescence and CBD yield (Atoloye et al. 2022; Farnisa et al. 2023; James et al. 2023; Ouyang et al. 2025; Short et al. 2021), limited information is available on the effects of organic amendments (Desai et al. 2024; Sicignano et al. 2025). In this study, dry inflorescence yield was highest under CM for both varieties (Spectrum, 1949 kg ha^− 1^; Therapy, 1320 kg ha^− 1^) when averaged over the years. These yields are lower than those reported under mineral N fertilization in other studies. James et al. (2023) estimated a dry inflorescence biomass yield of 3384 kg ha^− 1^ at an optimal N rate of 86.8 kg N ha^− 1^ in a field trial in North Carolina. However, our result is consistent with the findings of Bruce et al. (2022), who reported dry inflorescence yields of approximately 2,402 kg ha^− 1^ under compost treatment in a two-year organic hemp production study, though they remain below the yield potential under mineral N fertilization. The difference between the yield in our study compared to trials with mineral fertilizer is not unexpected given that compost was applied to supply 100 kg available N ha^− 1^. We assumed 15% of the N will be available in the first year, an approach which is consistent with mineralization estimates for stabilized plant-based composts (Geisseler et al. 2021). Although actual N availability during the growing season would have varied depending on soil temperature and moisture conditions, which differed substantially between years. Available N delivered by compost in any given season was probably lower than the 86.8 kg N ha^− 1^ that James et al. (2023) identified as yield-maximizing, which may partly explain the lower average yields observed in our study. At the same time, compost can supply benefits beyond N availability, including improvements in soil structure, water retention, and microbial activity, which may have sustained productivity at N supply levels that would otherwise be limiting under mineral fertilization alone (Adeleke et al. 2021).

Furthermore, in 2021, dry inflorescence yield in CC plots was the lowest of all treatments for both Spectrum (approximately 100 kg ha^− 1^) and Therapy (approximately 150 kg ha^− 1^). The low biomass was likely due to the combined effects of competitive suppression by cover crop regrowth and slow N release from surface-applied residues, which remained largely unavailable to the crop during the early growing season. In 2022, following the incorporation of cover crop residues into the soil, CC yields recovered substantially for both varieties, and CMCC produced the highest Spectrum yield in both years (3500 kg ha^− 1^). This yield was comparable to BAU synthetic N. We attribute this to the shift in termination method and the gradual accumulation of SOM and microbial biomass over time, each contributing to progressively greater organic N mineralization. Thus, the low CC and CMCC yields we observed in 2021 reflected the impact of the termination method rather than an inherent limitation of cover cropping as a management strategy. Moshay et al. (2024) noted that leguminous cover crops should ideally be terminated at 75% full bloom to maximize biomass production and avoid seed production, as continued maturity increases C: N ratios and decreases available nitrogen through remobilization to seed production. Thus, a proper cover crop termination method is critical for optimizing hemp performance, which is particularly important given the growing interest in sustainable soil management strategies to address plastic waste and soil health concerns in hemp production systems (Moshay et al. 2024; Osorio et al. 2023). Additionally, the higher yield under CMCC (Spectrum variety) appears greater than the additive effects of CM + CC alone. This demonstrates that combining compost and cover crops synergistically improves soil health more than their sole applications, and that this combination enhanced the yield in one of the varieties studied.

CBD yield is known to be positively correlated with dry inflorescence yield because CBD yield is calculated as the product of dry inflorescence yield and CBD percentage. The average CBD yield was highest under CM for both varieties (Spectrum, 173 kg ha^− 1^, Therapy 119 kg ha^− 1^). The observed average yields were lower than the CBD yield plateau of 389.34 kg ha^− 1^ reported by James et al. (2023) under mineral N fertilization at an optimal rate of 84.2 kg N ha^− 1^. While CBD yield generally follows the same trend as dry inflorescence yield, this was not true for Therapy in 2022, where the CBD yield between CC and CL was significantly different despite no differences in the dry inflorescence yield. This divergence in the Therapy variety’s response can be attributed to differences in CBD percentage. The CBD percent concentration for Therapy variety in 2022 followed the trend CC > CM = CMCC = CL. Previous studies have shown that CBD and THC percentages are primarily controlled by genetics and environmental factors, with fertility management playing a minimal role (Farnisa et al. 2023; Linder et al. 2022; Ouyang et al. 2025). While we observed amendment effects on cannabinoid content in one specific variety-year combination, this isolated occurrence requires additional research to determine whether it represents a reproducible response or a year-specific interaction. Elsewhere, Bruce et al. (2022) found that compost application yielded significantly higher CBD (8.82%) concentrations compared to premium-priced fertilizer blends, which produced the lowest concentrations (6.99% CBD). Panday et al. (2025) also found that organic N fertilization rates using blood meal affected the concentrations of CBD and THC in a study in Pennsylvania, suggesting that organic nutrient management strategies can influence cannabinoid profiles in hemp production systems.

Furthermore, regular THC monitoring enabled the detection of treatment-dependent differences in CBD content for the Therapy variety in 2022, which might have been missed had the plants been allowed to mature further (Bruce et al. 2022). Growers must balance maximizing CBD production with maintaining THC concentrations below the legal threshold of 0.3%, as high concentrations of CBD often coincide with elevated THC levels. This indicates that the window for optimal harvest may vary depending on soil amendment treatments and hemp varieties, emphasizing the need for precise monitoring of cannabinoid levels to maximize CBD yield while maintaining compliance with THC regulations. Additional work is needed to determine whether the observed amendment effects on cannabinoid content represent consistent varietal responses or isolated interactions, and if consistent, the mechanisms by which soil amendments influence hemp secondary metabolite production. Furthermore, the lack of amendment effect on CBD: THC ratio for Spectrum suggests that this variety maintains a stable cannabinoid profile regardless of fertility management, consistent with previous findings that cannabinoid ratios are primarily controlled by genetics (Campbell et al. 2019; Panday et al. 2025; Toth et al. 2021). The significant treatment effects observed for Therapy suggest that soil amendments may influence cannabinoid profiles in certain varieties under specific environmental conditions. This highlights the complex interactions between genetics, soil fertility, and environment in determining hemp cannabinoid composition.

### Amendment effect on hemp morphology and nutrient

Variety remained the dominant factor controlling plant height at both growth stages, with Spectrum consistently taller than Therapy. In 2021, Therapy height under CC (33 cm) was substantially lower than under CL (78 cm) at the late vegetative stage, which we attribute to competitive suppression from cover crop regrowth following incomplete crimping termination rather than N limitation, given that CL also received no N input. Overall, the minimal differences in the morphological attributes due to the soil amendments align with the results of Stack et al. (2023) who found that plant architecture was primarily variety-dependent. In addition, the lack of significant differences in leaf N values in 2022 likely reflects reduced mineralization across all treatments driven by lower July precipitation (128 mm vs. 143 mm in 2021), which would have reduced microbial activity and N release from residual soil organic matter, including in control plots (Huang and Hall 2017).

### Effects of amendments on soil health

The differential response of soil health indicators to amendments across years and varieties reflects the complex dynamics of soil ecosystems in agricultural settings. While previous studies have shown that organic matter inputs from compost and cover crops affect soil biological activity (Brennan and Acosta-Martinez 2019), our findings align with previous studies that have reported variable effects of soil amendments on biological and biochemical indicators (Agomoh et al. 2020; Oliveira et al. 2022). The significant differences observed in POXC, STBA, and CNPS in 2022 but not in 2021 suggest that the impact of amendments on these parameters may be cumulative or dependent on specific environmental conditions.

Soil organic carbon and TN accrued faster under compost than under cover crops alone (White et al. 2020). The larger gain under compost likely results from the direct input of stabilized, C- and N-rich organic matter, whereas cover-crop residues were returned in smaller amounts and contributed to SOC and TN more gradually (White et al. 2020). Although CMCC treatment received half the compost rate of CM (50 vs. 100 kg N ha^− 1^), it yet achieved an overall accrual rate intermediate between CM and the cover-crop and control treatments. Notably, the CMCC rate accelerated between years, an increase that coincided with the change in CMCC management from surface application of compost and cover-crop residue in 2021 to incorporation of both in 2022, suggesting that incorporation enhanced the contribution of the combined amendments to SOC. Because the cover-crop termination method changed between years rather than being held constant, this is a confounded management difference rather than a controlled comparison, and the second-year accrual rates were not statistically significant. The acceleration is therefore best interpreted as a trend consistent with an emerging compost-cover-crop interaction once incorporated, rather than a synergistic effect. In addition, the increase in SOC stocks above the baseline aligns with studies showing that hemp cultivation alone can enhance SOC. Jesmin et al. (2025) reported increased soil organic matter following cultivation of hemp grown for grain under N fertilization across subtropical Florida agroecosystems, attributing the gain to the crop’s fibrous, deep root system returning organic matter to the topsoil. This suggests that, beyond amendment inputs, the hemp crop itself contributed to the SOC accrual observed in this study.

### Leaf nutrient-yield relationships and soil health indicators

Leaf N concentration was positively correlated with both dry inflorescence and CBD yield at both vegetative stages, with stronger predictive power at mid-vegetative stage, consistent with Atoloye et al. (2022), who reported a similar positive relationship for floral hemp production in the same region. The stronger mid-vegetative relationship suggests that early-season N status is a more reliable indicator of yield potential than later-season measurements, likely because N availability during early establishment sets the trajectory for biomass accumulation. Also, the negative correlation between leaf P and yield parameters aligns with Westmoreland and Bugbee (2022), who found that floral hemp requires low P inputs, and elevated leaf P may reflect luxury uptake under conditions that are otherwise suboptimal for growth.

The stronger correlations between soil health indicators and agronomic parameters in 2021 than in 2022 likely reflect the greater soil N variability among treatments in the first year, when differences in amendment type produced more distinct soil N pools. In 2022, despite lower total July precipitation (128 mm vs. 143 mm in 2021), the higher number of rainy days (21 vs. 13) would have maintained more consistent soil moisture conditions throughout the mid-vegetative stage, enhancing mineralization across all treatments, including the control, and reducing treatment contrasts in leaf N and associated correlations. The shift toward biological indicators – specifically STBA and TOC – correlating with yield in 2022 suggests that as organic matter inputs accumulated, soil biological activity became a more relevant driver of productivity than mineral N availability alone. This is supported by the PCA, with CM and CMCC treatments grouping with biological soil health indicators at the late vegetative stage, while the year separation of data points confirmed that environmental conditions were a substantial source of variation independent of amendment treatment Khatri et al. (2025).

### Study limitations

Several limitations should be considered when interpreting the findings of this study. The cover crop termination method differed between years – crimping in 2021 and tillage incorporation in 2022 – which was a deliberate management adjustment rather than a controlled treatment factor. While this change likely contributed to the improved hemp performance observed in 2022, it introduces a confounding variable that limits direct year-to-year comparisons of cover crop treatment effects. Future studies should evaluate termination method as a treatment to better isolate its contribution to hemp productivity and soil nutrient dynamics. Bulk density was not directly measured in this study, and the SOC and TN stock estimates reported in Table 5 rely on an approximated value from the Web Soil Survey and should therefore be interpreted with caution. Furthermore, SOC and TN stock estimates were based on a small number of replicates per treatment and a single physically aggregated baseline, which limits the power to reliably detect and quantify short-term changes in SOC against large, spatially variable background stocks (Stanley et al. 2023). It is worth mentioning that while baseline SOC and TN stocks were from a physically aggregated sample, we cannot conclusively rule out the possibility that pre-existing spatial variability in C and N stocks contributed to the steep accrual observed in CM between baseline and 2021, although the research plots were not managed differently prior to the experiment, suggesting that the observed increases more likely reflect a “true” compost-driven response. Studies specifically aimed at tracking SOC change typically, therefore, incorporate replicated baseline sampling and an a priori power analysis to ensure detectable differences can be resolved (Müller et al. 2025; Stanley et al. 2023). The study was also conducted at a single location over two years, and the strong year effects observed suggest that environmental variability substantially shapes treatment responses. Multi-site, multi-year evaluations would strengthen the generalizability of these findings for hemp producers across the region.

## Conclusions

This research partially supported the hypothesis that compost and cover crop treatments would improve soil health and hemp biomass, although environmental factors could mask treatment effects. Organic soil amendments, including compost applied alone and in combination with cover crop, enhanced soil biological indicators, soil organic carbon and nitrogen stock, and improved hemp yield, though responses varied by variety and growing season. Spectrum variety consistently responded to compost applications with increased biomass and cannabidiol production, while Therapy showed variable responses independent of biomass changes. The unexpected finding that soil amendments affected cannabidiol concentration in one variety-year combination suggests that soil fertility may impact cannabinoid profiles, and this does not depend solely on genetics and environment. The response of fertility regimes was specific to variety, suggesting the need for continued research. Future studies should address the complex interactions between soil amendments, environmental factors, and genetic variability to optimize cannabidiol production in hemp cultivation.

## Supplementary Information

Supplementary Material 1.

## Data Availability

The datasets used and/or analyzed during the current study are available from the corresponding author on reasonable request.

## Abbreviations

CBD: Cannabidiol
CL: Control
CM: Compost
CC: Cover crop
CMCC: Compost and cover crop
THC: Tetrahydrocannabinol
DAT: Days after transplanting
EC: Electrical conductivity
SOC: Soil organic carbon
TOC: Total organic carbon
TN: Total nitrogen
NO₃⁻: N-Nitrate nitrogen
NH₄⁺: N-Ammonium nitrogen
POXC: Permanganate oxidizable carbon
STBA: Soil test biological activity
DHA: Dehydrogenase
CNPS: Carbon, nitrogen, phosphorus, and sulfur
TTC: Triphenyl tetrazolium chloride
PCA: Principal component analysis
