# Supplementary material for: The effect of organic amendments on cannabidiol yield and topsoil health (0–10 cm) in floral hemp (Cannabis sativa L.) production system
Source: J Cannabis Res. 2026 Aug 14;8:122. doi: 10.1186/s42238-026-00485-x (PMC13753258; doi:10.1186/s42238-026-00485-x)
Supplement: Supplementary file 1 [file 42238_2026_485_MOESM1_ESM.docx]

Supplementary tables and figures

Table S1 Weather conditions with average monthly precipitation, total monthly precipitation, and average daily temperature for the growing seasons of the study from the weather station operated by North Carolina State Climate Office closest to the study site in Greensboro, NC (36° 3' N, 79° 44' W).

| Year | Month | Average Precipitation (mm) | Total Precipitation (mm) | Number of rainy days | Average Temperature (ºC) |
| --- | --- | --- | --- | --- | --- |
| 2021 | June | 3.97 | 119 | 13 | 23.2 |
|  | July | 4.62 | 143 | 13 | 25.1 |
|  | August | 3.70 | 115 | 12 | 25.2 |
|  | September | 4.22 | 126 | 6 | 21.4 |
|  | October | 2.20 | 68 | 9 | 17.5 |
| 2022 | June | 2.24 | 67 | 7 | 24.3 |
|  | July | 4.12 | 128 | 21 | 25.5 |
|  | August | 2.74 | 85 | 12 | 24.3 |
|  | September | 4.39 | 132 | 10 | 20.8 |
|  | October | 1.53 | 48 | 8 | 13.7 |

Table S2. Cannabinoid profiles of the amendment trial in 2021 and 2022 at harvest.

| Year | Variety | Soil Amendment | CBD | CBDA | CBDV | Total CBD | Δ9 THC | THCA | THCV | Total THC | CBG | CBGA | CBC | Total Cannabinoids |
| --- | --- | --- | --- | --- | --- | --- | --- | --- | --- | --- | --- | --- | --- | --- |
| 2021 | Spectrum | CL | 1.07 | 10.7 | - | 10.5 | 0.12 | 0.37 | - | 0.44** | 0.08 | 0.36 | 0.11 | 12.8 |
|  |  | CM | 1.09 | 8.92 | - | 8.9 | 0.12 | 0.31 | - | 0.38** | 0.07 | 0.27 | 0.11 | 10.9 |
|  |  | CC | 1.22 | 9.65 | - | 9.68 | 0.13 | 0.33 | - | 0.41** | 0.08 | 0.33 | 0.11 | 11.8 |
|  |  | CMCC | 1.00 | 8.81 | - | 8.72 | 0.10 | 0.30 | - | 0.36** | 0.06 | 0.28 | 0.10 | 10.6 |
|  | Therapy | CL | 3.55 | 6.5 | 0.03 | 9.25 | 0.62* | 0.27 | 0.01 | 0.85a** | 0.1 | 0.19 | 0.25 | 11.5 |
|  |  | CM | 3.07 | 6.85 | 0.01 | 9.08 | 0.62 | 0.46 | 0.01 | 1.01a** | 0.09 | 0.21 | 0.25 | 11.6 |
|  |  | CC | 2.74 | 6.89 | 0.04 | 8.78 | 0.21 | 0.16 | 0.01 | 0.35b** | 0.07 | 0.18 | 0.19 | 10.5 |
|  |  | CMCC | 3.09 | 7.77 | 0.02 | 9.9 | 0.24 | 0.18 | - | 0.39b** | 0.1 | 0.26 | 0.21 | 11.9 |
| 2022 | Spectrum | CL | 7.40 | 0.47 | - | 7.81 | 0.24 | - | - | 0.24 | 0.23 | 0.06 | 0.44 | 8.85 |
|  |  | CM | 6.73 | 0.57 | - | 7.23 | 0.23 | - | - | 0.23 | 0.17 | 0.06 | 0.46 | 8.17 |
|  |  | CC | 6.85 | 0.51 | - | 7.30 | 0.23 | 0.02 | - | 0.24 | 0.19 | 0.05 | 0.42 | 8.23 |
|  |  | CMCC | 7.13 | 0.48 | - | 7.55 | 0.24 | - | - | 0.24 | 0.17 | 0.05 | 0.5 | 8.55 |
|  | Therapy | CL | 4.29 | 0.25 | - | 4.51b | 0.99* | - | - | 0.99** | 0.19 | - | 0.2 | 5.93 |
|  |  | CM | 5.29 | 0.23 | - | 5.49ab | 0.71* | - | - | 0.71** | 0.19 | 0.04 | 0.24 | 6.67 |
|  |  | CC | 7.74 | 0.38 | 0.16 | 8.07a | 1.02* | - | - | 1.02** | 0.29 | 0.06 | 0.42 | 9.94 |
|  |  | CMCC | 4.95 | 0.28 | 0.11 | 5.2b | 0.83* | - | - | 0.83** | 0.18 | - | 0.23 | 6.51 |

All values are in percent (%) of dry mass. An empty value indicates that the cannabinoid could not be detected with <0.001 % accuracy. The total cannabinoids are the sum of total THC, total CBD, and minor cannabinoids. An asterisk (*) next to the Δ9 THC concentration indicates that it was above the 0.3% threshold. Two asterisks (**) next to the total THC concentration indicates it was above the 0.3% threshold. CL, control; CM, compost; CC, cover crop only; CMCC, compost and cover crop. THC, tetrahydrocannabinol; CBD, cannabidiol; CBDA, cannabidiolic acid, CBDV, cannabidivarin; THCA, tetrahydrocannabinolic acid; THCV, tetrahydrocannabivarin; CBG, cannabigerol; CBGA, cannabigerolic acid; CBC, cannabichromene. In 2021, Therapy was harvested on October 14 and Spectrum on October 21, while in 2022 Spectrum was harvested on September 29 and Therapy on October 12.

Table S3: Effect of amendments on soil properties in Year 2021 and 2022.

|  |  |  | Year 2021 | | Year 2022 | |
| --- | --- | --- | --- | --- | --- | --- |
| Variety | Stage | Amendment | pH | EC (µS cm^-1^) | pH | EC (µS cm^-1^) |
| Spectrum | MV | CL | 7.6(0.1)a | 137(17)b | 7(0.09) | 186(23)b |
|  |  | CM | 6.9(0.11)b | 531(52)a | 6.9(0.16) | 354(42)a |
|  |  | CC | 7.3(0.24)ab | 120(16)b | 6.6(0.34) | 184(10)b |
|  |  | CMCC | 7.3(0.02)ab | 140(18)b | 7(0.02) | 266(45)ab |
|  | LV | CL | 7.5(0.05) | 144(35)b | 7.5(0.06)a | 56(8)bc |
|  |  | CM | 7.2(0.19) | 258(28)a | 6.9(0.2)b | 154(34)a |
|  |  | CC | 7.3(0.23) | 92(9)b | 6.9(0.29)b | 50(6)c |
|  |  | CMCC | 7.4(0.07) | 137(4)b | 6.9(0.17)b | 86(25)b |
| Therapy | MV | CL | 7.5(0.05)a | 148(6)b | 7.2(0.08) | 180(11)b |
|  |  | CM | 6.9(0.25)b | 313(21)a | 6.9(0.08) | 292(36)a |
|  |  | CC | 7.4(0.11)a | 94(14)c | 6.7(0.2) | 180(12)b |
|  |  | CMCC | 7.3(0.17)ab | 134(8)bc | 7(0.08) | 218(9)ab |
|  | LV | CL | 7.3(0.15) | 188(69)a | 7.5(0.09)a | 96(9)b |
|  |  | CM | 7.1(0.21) | 184(24)a | 6.9(0.09)b | 193(30)a |
|  |  | CC | 7.4(0.2) | 96(11)b | 7.1(0.11)ab | 82(7)b |
|  |  | CMCC | 7.3(0.13) | 126(1)ab | 7.1(0.19)ab | 102(21)b |
| Amendment (A) | | | *** | *** | ** | *** |
| Variety (V) | | | ns | ns | ns | ns |
| Stage (S) | | |  |  |  |  |
| A × V | | | ns | ns | ns | ns |
| A × S | | |  |  |  |  |
| S × V | | |  |  |  |  |

^The data represent the mean (standard error); Different lowercase letters indicate significant differences among amendments within the same stage (linear mixed-effect model and Tukey’s HSD; n=3). * indicates significant at the 0.05 probability level, **Significant at the 0.01 probability level. ***Significant at the 0.001 probability level, ns indicates non-significant. - data not collected. MV, mid-vegetative; LV, late-vegetative; CL, control; CM, compost; CC, cover crop only; CMCC, compost and cover crop; EC, electrical conductivity.^

Figure S1: Correlation of soil health indicators with plant agronomic attributes. MV, mid-vegetative stage; LV, late vegetative stage; CBD, cannabidiol; DB, dry inflorescence; N, nitrogen; Moist, gravimetric moisture; EC, electrical conductivity; TOC, total organic carbon; TN, total nitrogen; NO_3_ nitrate; NH_4_, ammonium; POXC, permanganate oxidizable carbon; STBA, soil test biological activity; DHA dehydrogenase enzyme activity; CNPS, carbon, nitrogen, phosphorus and sulfur.

a

b

c

d
